# Incest versus abstinence: reproductive trade-offs between mate limitation and progeny fitness in a self-incompatible invasive plant

**DOI:** 10.1002/ece3.875

**Published:** 2013-11-18

**Authors:** Jennifer C Pierson, Stephen M Swain, Andrew G Young

**Affiliations:** CSIRO Plant IndustryGPO Box 1600, Canberra, ACT, 2601, Australia

**Keywords:** Baker's rule, biparental inbreeding depression, genetic load, mate availability, *Raphanus raphanistrum*, reproductive assurance, sporophytic self-incompatibility

## Abstract

Plant mating systems represent an evolutionary and ecological trade-off between reproductive assurance through selfing and maximizing progeny fitness through outbreeding. However, many plants with sporophytic self-incompatibility systems exhibit dominance interactions at the *S*-locus that allow biparental inbreeding, thereby facilitating mating between individuals that share alleles at the *S*-locus. We investigated this trade-off by estimating mate availability and biparental inbreeding depression in wild radish from five different populations across Australia. We found dominance interactions among *S*-alleles increased mate availability relative to estimates based on individuals that did not share *S*-alleles. Twelve of the sixteen fitness variables were significantly reduced by inbreeding. For all the three life-history phases evaluated, self-fertilized offspring suffered a greater than 50% reduction in fitness, while full-sib and half-sib offspring suffered a less than 50% reduction in fitness. Theory indicates that fitness costs greater than 50% can result in an evolutionary trajectory toward a stable state of self-incompatibility (SI). This study suggests that dominance interactions at the *S*-locus provide a possible third stable state between SI and SC where biparental inbreeding increases mate availability with relatively minor fitness costs. This strategy allows weeds to establish in new environments while maintaining a functional SI system.

## Introduction

Theory predicts that self-incompatible (SI) species will be reproductively and demographically challenged when colonizing new habitats. This is because mate availability, or the number of individuals with compatible mating genotypes, can be severely limited in small founding populations (Baker 1974; Pannell and Barrett 1998; Dornier et al. 2008). Several studies of mating systems in weeds support the theory that self-compatible (SC) species may be more successful invaders (Price and Jain 1981; Barrett 1992; Rambuda and Johnson 2004; Van Kleunen et al. 2008). For example, Hao et al. (2010) found a higher than expected proportion of self-compatible invasive Asteraceae species in China. Paradoxically, several highly successful invasive species have SI mating systems (Elam et al. 2007; Abbott et al. 2008). This raises the question of how SI plants can successfully overcome the mate limitation faced when long-distance dispersal is combined with very small founding population sizes.

One hypothesis is that SI plants in these circumstances evolve toward SC due to intense selection for reproductive assurance during the colonization and establishment phase (Busch and Schoen 2008; Busch and Delph 2011; Petanidou et al. 2012). Mating systems are an area of rapid evolution during the invasion process (Barrett et al. 2008) as selective pressure for reproductive assurance is high in small founding populations. Some of this rapid evolution may be adaptive (Billiard et al. 2006; Brennan et al. 2010), and some may be due to drift associated with founder effects. Most SI species experience some levels of SC, often termed pseudo-self-compatibility (PSC) due to a leaky response in the SI system (Levin 1996) and therefore have the opportunity to evolve toward SC. The conversion of a plant's mating system from SI to SC can be an adaptive advantage in the short term if the fitness cost due to inbreeding (inbreeding depression) does not negate the advantages of reproductive assurance. Generally, if the fitness cost of being SC is <50% that of being outcrossed, then selection will drive the population toward SC (Busch and Delph 2011). In the long term, plants may benefit from maintaining SI as outcrossed populations generally enjoy higher fitness than inbred populations, even after purging (Petanidou et al. 2012). It is uncommon to evolve from SC to SI (Igic et al. 2008) and therefore appears to be very difficult for plants to “regain” SI once lost. Many plants have a mixed mating system (between 20 and 80% outcrossing; Lande and Schemske 1985) which was thought to represent the transition between SI and SC (Lande and Schemske 1985; Goodwillie et al. 2005). However, the frequency of mixed mating in plants has led to the hypothesis that mixed mating is an evolutionary stable strategy (Goodwillie et al. 2005; Winn et al. 2011).

An alternative hypothesis for how SI plants overcome mate limitation during colonization is that biparental inbreeding may increase mate availability, relative to incompatibility among all related individuals, enough to overcome Allee effects. Biparental inbreeding is mating among related individuals. Self-incompatibility is controlled by a group of tightly linked genes, the *S*-locus, and fertilization is prevented by an SI response that is activated when individuals with shared alleles interact. There are two major types of SI, gametophytic (GSI), and sporophytic (SSI) (Busch et al. 2012). In GSI, alleles are codominant, whereas alleles can be dominant, codominant, or recessive in the SSI system. Male gametophytes from SSI plants express the parental diploid phenotype and often exhibit dominance that allows some mating among individuals with a single shared allele (Brennan et al. 2010) which may increase mate availability relative to an SI system without dominance (Brennan et al. 2003). Matings permitted among relatives due to dominance among *S*-alleles may represent an intermediate option between self-incompatibility and self-compatibility. This is fundamentally different from a mixed mating strategy in which both self-fertilization and outcrossing occur regularly (Winn et al. 2011). Dominance interactions that allow biparental inbreeding may be a viable strategy to increase mate availability in the short term to allow successful colonization of sites after long-distance dispersal events such as invasions (Brennan et al. 2003, 2010; Lafuma and Maurice 2007) without forfeiting the long-term fitness benefits of maintaining a SI mating system.

However, biparental inbreeding typically incurs a fitness cost in the form of reduced viability or fecundity (biparental inbreeding depression; Nason and Ellstrand 1995; Wagenius et al. 2010). For SC to be an evolutionarily stable strategy, this cost typically needs to be less than half the fitness of outcrossed individuals (Lande and Schemske 1985). In many highly outcrossing species, the fitness cost may be such that inbreeding is not a viable option, as the population cannot persist long enough to sufficiently purge deleterious alleles.

We set out to empirically test the hypothesis that biparental inbreeding due to mating between individuals sharing intermediate dominance alleles may represent a strategy to increase mate availability in a highly successful globally invasive plant, wild radish (*Raphanus raphanistrum*). Specifically, we explored the trade-off between increased mate availability and fitness cost by asking the following questions: (1) Is there evidence that dominance among *S*-alleles facilitates mating among related individuals? (2) Does biparental inbreeding result in a significant increase in mate availability? and (3) What is the fitness cost of biparental inbreeding?

## Materials and Methods

### Study system

Wild radish is a globally invasive plant that is a good model system in which to investigate these questions. It is an annual Brassicaceae that has a SSI mating system with complex dominance interactions at the *S*-locus and is highly outcrossing (Sampson 1964). In the close relative, *R. sativus*, biparental inbreeding and biparental inbreeding depression occur, and the latter has been shown to reduce reproductive output when population sizes are small (Allee effect; Elam et al. 2007). Both species have been well studied, so there is considerable information known about their life history and invasive success.

*Raphanus raphanistrum* is native to the Mediterranean area and has successfully invaded most regions of the world (Holm et al. 1997). It is a common agricultural weed and is increasingly becoming an environmental weed and hybrid with *R. sativus* (Ridley and Ellstrand 2008). A likely reason this annual herb has been so successful is that it can produce hundreds of flowers and >15,000 seeds/m^2^ and established populations are characterized by a dense seed bank that can persist for five to ten years (Reeves et al. 1981).

We estimated rates of mate availability, due to outcrossing and dominance interactions, and biparental inbreeding depression in plants from five populations across Australia. As rates of mate availability and biparental inbreeding depression may vary in natural populations, seeds sourced from five wild populations across Australia were included to capture some of this variation. Two populations were in Western Australia: one approximately 100 km NE of Perth: PG4 (31°24′S, 116°19′E) and one in Albany: AL (34º49′S, 117°58′E). Two populations were in South Australia: one in Auburn: SA1 (34°05′S, 138°41′E) and one in Mallala: SA4 (34°45′S, 138°50′E). The fifth population was located in Tylden, Victoria: VIC3 (37°20′S, 144°23′E).

### Mate availability

Complete diallel crosses were conducted among 15 individuals per population to estimate mate availability and determine the sharing frequency of intermediate dominance *S*-alleles. Intermediate dominance alleles result in one-way compatibility that is readily detected using diallel crosses. Seeds from each population were grown in a glasshouse and hand-pollinations were conducted to cross each individual with each other as both the female and the male parent to test for compatibility in both directions. Each plant was also self-pollinated. All crosses were conducted on freshly opened flowers that were bagged following pollination. Open-pollinated controls were left on each plant in the glasshouse to check that pollinators were being effectively controlled in the glasshouse.

Dominance interactions at the *S*-locus will result in crosses that are successful in one direction. Pollen expresses the diploid phenotype of the parent and the stigma only expresses the dominant haploid phenotype which allows unidirectional compatibility among plants. Mate availability among individuals without shared alleles was estimated by counting the proportion of matings that were cross-compatible in both directions (male/female). The proportion of mate availability due to matings among individuals with shared alleles was estimated by counting the proportion of matings that were cross-compatible in only one direction.

### Biparental inbreeding

**Figure 1:**
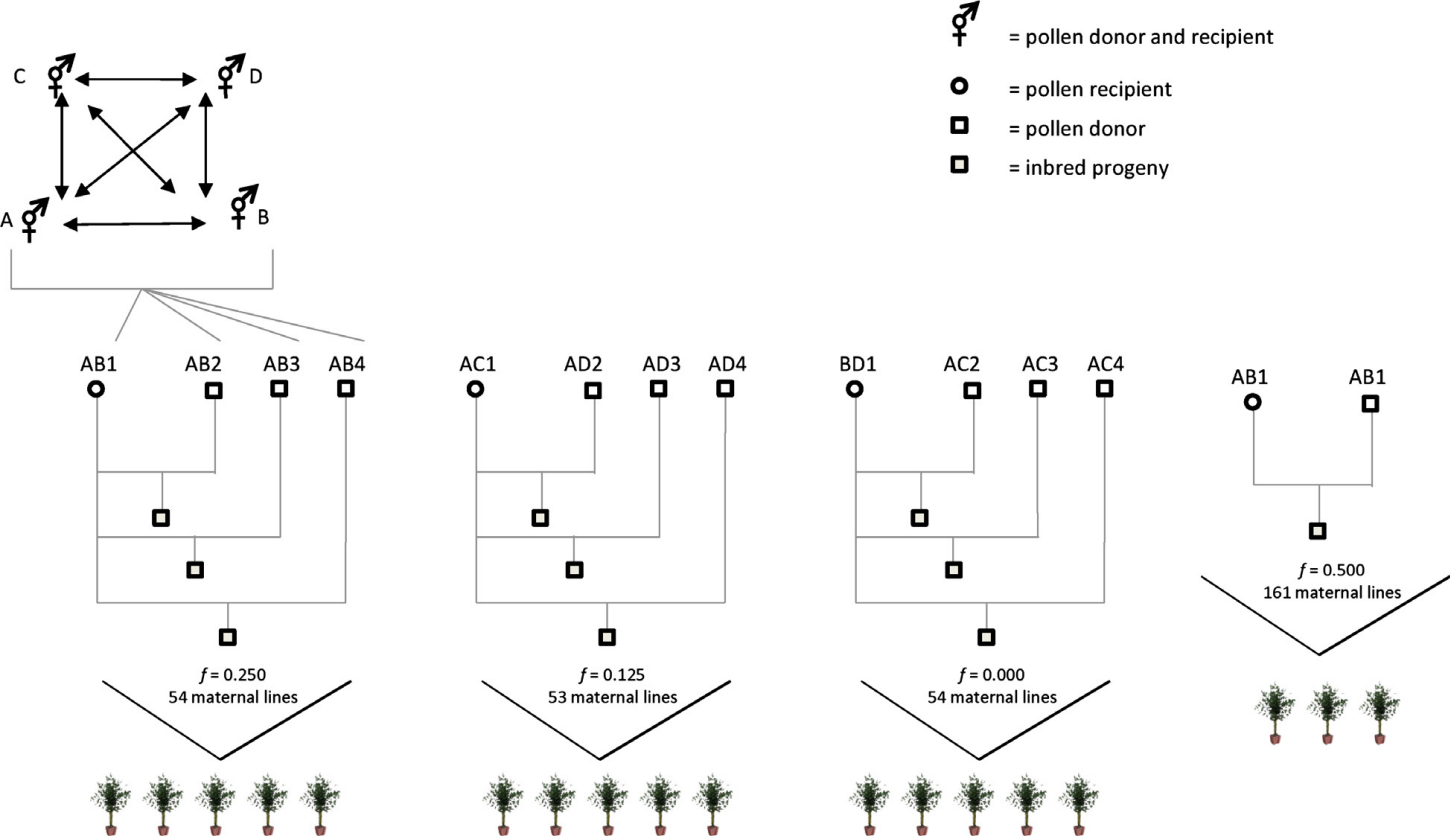
Four levels of inbreeding were created from wild collected seeds and the inbred progeny were germinated and grown in an outdoor enclosure protected from birds but open to natural pollinators. In each population, 2 sets of four fully compatible individuals were identified using full diallel crosses (top left) and the progeny of these crosses was used to create full-siblings (*f* = 0.250), half-siblings (*f* = 0.125), and unrelated progeny. For each crossing type, a maternal plant was pollinated by three replicates of a pollen donor and each cross was made three times. Each maternal plant was also selfed (*f* = 0.500). The seeds from each maternal line cross-type (e.g., AB1 full-siblings) were pooled, and five seeds were randomly chosen to be germinated in the outdoor experiment.

We used the results from the intrapopulation diallel crosses to identify two sets of four individuals from each population that were fully cross-compatible, indicating they shared no *S*-alleles and could be considered unrelated. The seeds from these crosses were used to create a range of cross-types including unrelated individuals (UR), half-siblings (HS), full-siblings (FS), and selfs (SC) (Table 1, Fig. 1). This was performed using a two-generation controlled cross-experiment. Specifically, four seeds each from each maternal line of compatible cross were randomly selected, and seeds were nicked and germinated in petri dishes and then transferred to pots in a greenhouse that was essentially free of pollinators. For example, if plant A and plant B were compatible (AB), then four seeds that resulted from this cross were selected for germination (e.g., AB1, AB2, AB3, and AB4). Each maternal line was then used to pollinate three replicates of a particular cross-type. For instance, if AB was used to create full-siblings, AB1 would be pollinated by AB2, AB3, and AB4 (Fig. 1). A selected newly opened flower was visually checked for pollen, hand-pollinated, and covered with a small envelope. Controls were set up in the same way but without pollination. Each plant was also self-pollinated. Each parental combination/control was replicated three times resulting in 1,563 hand-pollinations and 328 controls (Table 1).

**Table 1:** The number of maternal lines, hand-pollination crosses (pollinations), seeds germinated (seed), and seedlings transplanted to the outdoor enclosure per cross-type (UR = unrelated *f* = 0, HS = half-sibling, *f* = 0.125; FS = full-sibling, *f* = 0.250; SC = selfed individual, *f* = 0.500).

Cross-type	Maternal lines	Pollinations	Seed	Outdoor enclosure
UR	54	419	260	177
HS	53	469	207	151
FS	54	469	198	121
SC	161	206	62	24

Crosses were considered successful if seed set occurred after hand-pollinations. Seeds were collected from successful crosses to be grown outdoors in pots in an enclosure that excluded birds but allowed open-pollination. We randomly chose five seeds/cross-type from each maternal line (i.e., AB1 x AB2, AB3, AB4) to germinate for a total 727 seeds from 141 maternal lines (Table 1). Because not all the crosses produced seed, there was an uneven number of seeds/cross-type (treatment; Table 1). Seeds were individually weighed and then planted in jiffy pots on 27 May 2011 and kept in outdoor cold frames that were covered at night. This precaution was taken because Canberra, Australia, experiences freezing temperatures at night more often than many of locations from which the seeds were sourced. The jiffy pots were watered daily for 30 days and germinated seedlings were transferred to the outdoor enclosure. On 6 July 2011, 473 seeds had germinated and were planted in 20-cm-diameter pots containing one-third potting mix, one-third sand, and one-third peat moss. A randomized design was used to place plants in a 20–24 grid within the enclosure. Plants were exposed to natural conditions and watered for 2 min every 2 days by an overhead sprinkler system.

We recorded a variety of phenological, growth, and reproductive variables to represent critical life-history stages (Table 2). Plants were checked every other day to record the first day of bolting, first day of flowering, and death. Plant growth characteristics, rosette width and number of leaves, were measured on the same day for all plants. Plant width was measured as the diameter at the widest point of the rosette, and the number of leaves in the rosette was counted. All surviving plants were harvested between 31 October and 4 November 2011. Plant height was measured from the base of the plant (soil surface) to the top of the tallest stem, and roots were washed and dried to be weighed. Aboveground plant material was dried at room temperature prior to estimating reproductive output, and measuring aboveground plant weight and seedpod weight.

**Table 2:** Results of REML analysis testing whether cross-type (UR, HS, FS, UR), and the interaction between cross-type and population significantly explains variation in pregermination, growth, phenological, and reproductive output parameters. Survival was universally high >97% and was excluded from the analyses. Variables in bold are discussed in detail in the results section.

	Cross-type	Cross-type*pop
Pregermination		
**Seed-set rate**	*D*_3_ = 164.64**	*D*_12_ = 34.07
Seed weight (square root)	*F*_3_ = 5.4*	*F*_12_ = 1.19
Growth		
**Aboveground biomass** (square root)	*F*_3_ = 14.04**	*F*_10_ = 1.31
Plant height	*F*_3_ = 13.6**	*F*_10_ = 0.74
Number of apical stems (square root)	*F*_3_ = 9.49**	*F*_12_ = 0.87
Rosette width (square root)	*F*_3_ = 8.19**	*F*_10_ = 0.64
Number of leaves (square root)	*F*_3_ = 6.48**	*F*_12_ = 0.69
Dried root weight (square root)	*F*_3_ = 11.43**	*F*_12_ = 0.86
Phenology		
Days germination to bolt	*F*_3_ = 2.36	*F*_10_ = 1.18
Days germination to flowering	*F*_3_ = 1.79	*F*_10_ = 1.33
Days bolting to flowering	*F*_3_ = 1.88	*F*_10_ = 1.27
Reproductive output		
**Seedpod weight** (square root)	*F*_3_ = 10.34**	*F*_10_ = 1.03
Estimated number of seeds (square root)	*F*_3_ = 7.79**	*F*_10_ = 0.36
Estimated number of fattened pedicles (square root)	*F*_3_ = 7.83**	*F*_10_ = 0.32
Estimated number of pedicles (square root)	*F*_3_ = 10.78**	*F*_10_ = 0.78
Flower to pod conversion rate	*F*_3_ = 1.81	*F*_10_ = 0.19

* *P* < 0.01,

** *P* < 0.001.

Reproductive output was estimated by counting the number of apical stems on each plant, and subsampling 20 stems on which the number of total pedicles (representing flowers) and fattened pedicles (representing pods that contained seeds) were counted. Ten seedpods were randomly selected and the number of seeds per pod was recorded. The number of flowers was estimated by multiplying the mean number of flowers per stem by the number of stems, and the number of fattened pods was estimated by multiplying the mean number of fattened pedicles per stem by the number of stems. Finally, all the seedpods were removed from the plant and weighed to measure total seedpod weight.

### Statistical analysis

Although a randomized design was used to place plants in the enclosure, it is possible that natural variation in the microclimate of the enclosure could have influenced plant growth, survival, or reproductive output. Therefore, we examined individual plant responses for each variable of interest using heat maps where values are represented by different colors which allows visual analysis of spatial patterns in the data (created in R version 3.0.1; R core team. 2013). As none of the variables appeared to have spatial patterns, we did not include spatial location in the subsequent analysis.

Seed set was a binary variable that represented a positive (at least one seed set) or negative (no seeds) response to pollination per each mom/dad combination. A binomial generalized linear model with a logit link was used to estimate seed-set rate per cross-type. For all other variables considered, we used linear mixed models that were fitted using the restricted maximum-likelihood approach (REML) to account for unbalanced data (Table 1). In all models, mother was included as a random factor, while cross-type, population, and the interaction between cross-type and population were included as fixed factors. Diagnostic plots of the residuals were checked for normality, and data were transformed when necessary to meet normality assumptions (Table 2). Standard errors were calculated on back-transformed variables, and standard error of the differences (SED) was used to test for significance between means. Analyses were performed in GenStat (Payne et al. 2009).

### Inbreeding depression

Inbreeding depression was calculated for each level of inbreeding (*f* = 0.125, *f* = 0.250, *f* = 0.500) using the following equation (Keller and Waller 2002):


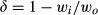


where *w*_*i*_ = fitness of inbred progeny and *w*_*o*_ = fitness of outbred progeny. Inbreeding depression was calculated for rate of seed-set, plant biomass, and seedpod weight as these variables represented typical responses within their respective life-history categories (Table 2). Cumulative inbreeding depression was calculated by multiplying mean relative fitness of pregermination and postgermination life-history stages (seed-set rate * seedpod weight) (Husband and Schemske 1996). Genetic load, measured as lethal equivalents, was estimated using the linear regression approach using the following equation:


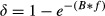



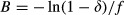


where B = genetic load per gamete and *f* = inbreeding coefficient. The number of lethal equivalents (LE) is 2B for diploid individuals (Keller and Waller 2002).

## Results

### Mate availability

**Figure 2:**
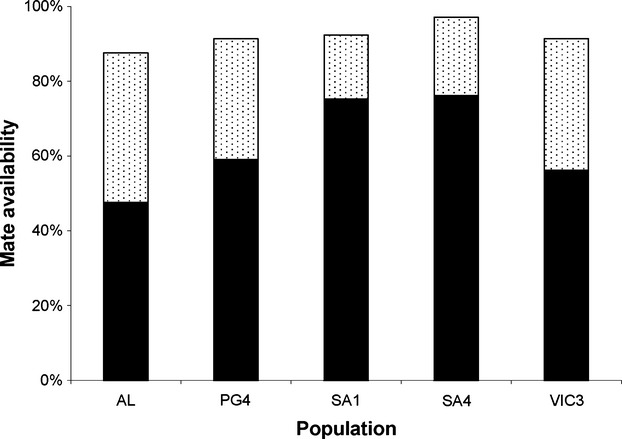
Estimates of mate availability based on complete diallel crosses of 15 individuals per population. Solid black bars indicate the percent of mate availability due to fully compatible individuals, and the dotted bars indicate the percent of mate availability due to one-way compatible individuals, indicating a shared allele at the *S*-locus.

Total mate availability (MA) was consistently high across all populations and ranged from 0.88 to 0.97 (Fig. 2). The proportion of MA due to fully compatible crosses ranged from 0.48 to 0.76. The proportion of MA due to dominance interactions between *S*-alleles ranged from 0.17 to 0.40 (Fig. 2).

### Biparental inbreeding depression

We tested 16 variables in four life-history categories, pregermination, growth, phenology, and reproductive output, for significant effects of cross-type, and the interaction between cross-type and population (Table 2). Twelve of the 16 variables showed a significant effect of cross-type; however, none of the variables showed a significant effect for the interaction between cross-type and population (Table 2). Consequently, source population was eliminated from further analyses, as was survival due to high overall values (>97%).

### Pregermination

**Figure 3:**
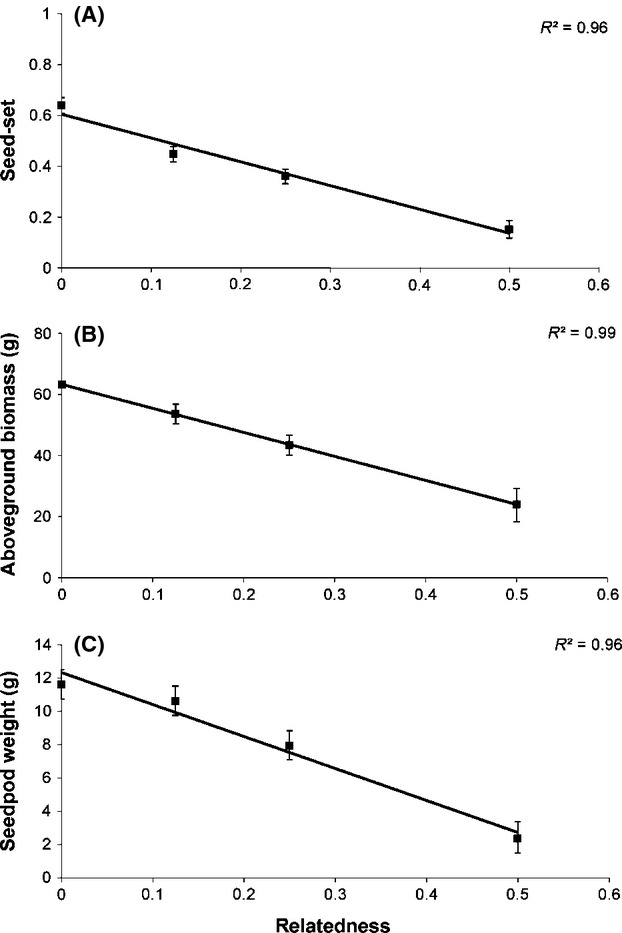
The relationship between genetic relatedness (inbreeding coefficient *f*) and predicted means of fitness variables for three different life-history stages: (A) pregermination (seed-set rate), (B) growth (aboveground biomass back-transformed), and (C) reproductive output (seedpod weight back-transformed). Correlation estimates (adjusted *R*^2^) were obtained from linear regression in the statistical software R, version 3.0.1 (R core team. 2013).

**Figure 4:**
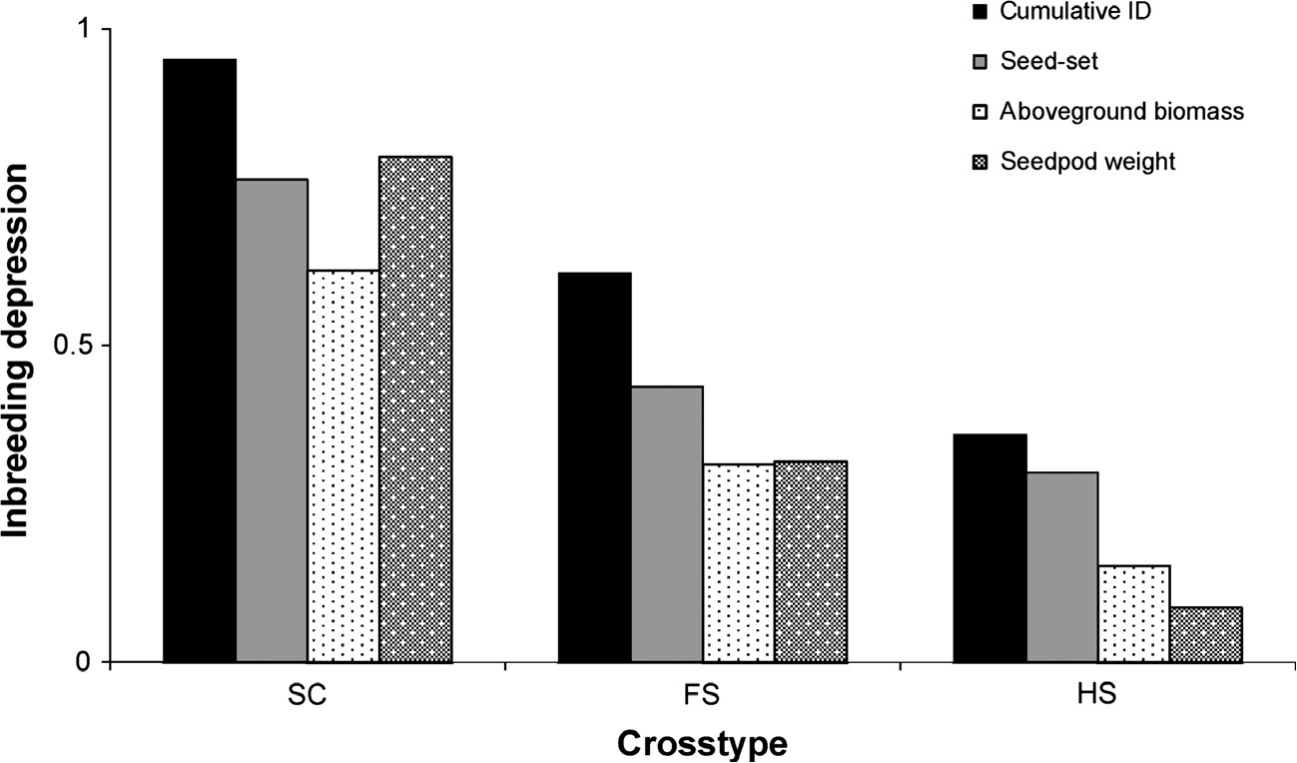
Estimates of inbreeding depression, cumulative and per life-history stage, for selfed plants (SC), full-siblings (FS), and half-siblings (HS). Cumulative ID was calculated by multiplying means per cross-type of seed-set rate * seedpod weight.

Seed-set and seed weight varied significantly by cross-type (*P* < 0.001; Table 2), but the interaction between cross-type and population was not significant at α = 0.05 level. However, variation in seed-set among populations did appear to exist and was significant at α = 0.10 level (*P* = 0.09). Seed-set rate is a good representative of pregermination responses as it encompasses both fertilization and seed abortion rate to give a metric of successful seed production. Therefore, we present specific results for this variable only. Seed set had a strong negative linear relationship (*R*^2^ = 0.97) with the level of genetic relatedness (Fig. 3a) with estimates of inbreeding depression (ID) ranging from 0.76 for selfed individuals (*f* = 0.500) to 0.30 for half-siblings (*f* = 0.125; Fig. 4). The genetic load or number of lethal equivalents (LE) estimated to be associated with seed-set rate was 5.71 for selfed individuals, 4.57 for full-siblings, and 5.68 for half-siblings (Table 3).

**Table 3:** The estimated number of lethal equivalents per cross-type for cumulative ID and representative variables of three life-history stages.

	Cumulative ID	Seed-set rate	Aboveground biomass	Seedpod weight
Selfed	11.98	5.71	3.87	6.44
Full-sibling	7.62	4.57	3.00	3.05
Half-sibling	7.11	5.68	2.64	1.43

### Postgermination

Three phenological variables were evaluated, none of which varied significantly by cross-type or for the interaction between cross-type and population. All six growth variables significantly varied by cross-type (*P* < 0.001; Table 2), with level of inbreeding showing a consistently negative effect on *R. raphanistrum* growth. Aboveground biomass is a good indicator of overall growth as it tends to reflect plant height, number of stems, and rosette size all of which result in higher biomass; therefore, we present specific results for this growth variable only. Biomass had a strong negative linear relationship with the level of genetic relatedness (*R*^2^ = 0.99; Fig. 3b). Estimates of ID ranged from 0.62 for selfed individuals to 0.15 for half-siblings (Fig. 4), resulting in an estimated 3.87 LE in selfed individuals to 2.64 LE in half-siblings (Table 3).

Four of the five reproductive output variables varied significantly by cross-type (*P* < 0.001; Table 2), again displaying a consistent negative relationship between reproductive output and inbreeding. Seedpod weight is a good estimate of maternal fecundity as it encompasses both seed-set and seed size and was measured directly (number of flowers, seedpods, and seeds were estimated by subsampling). Therefore, we present specific results for this reproductive output variable only. Seedpod weight had a strong negative linear relationship with the level of genetic relatedness (*R*^2^ = 0.97; Fig. 3c). Estimates of ID ranged from 0.80 for selfed individuals to 0.09 for half-siblings (Fig. 4), resulting in an estimated 6.44 lethal equivalents in selfed individuals to 1.43 LE in half-siblings (Table 3).

Cumulative inbreeding depression was considered as the product of pregermination and postgermination fitness and was calculated as the product of seed-set rate * seedpod weight. Cumulative ID estimates were 0.95, 0.61, and 0.36 for selfed individuals, full-siblings, and half-siblings, respectively (Fig. 4) and LE ranged from 11.98 to 7.11 for selfed plants to half-siblings (Table 3).

## Discussion

**Figure 5:**
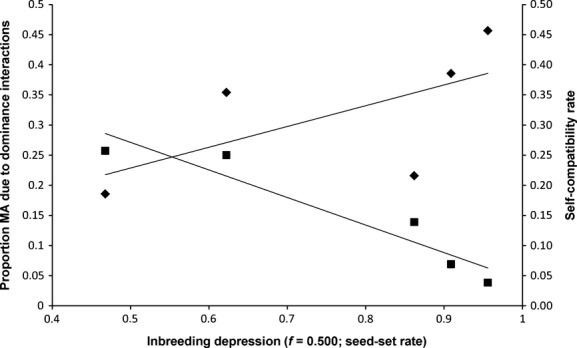
The relationship between the proportion of mate availability (MA; left axis) due to dominance interactions at the *S*-locus and estimates of inbreeding depression based on selfed individuals (*f* = 0.500; seed-set rate) in five populations of *R. raphanistrum* (*R*^2^ = 0.62; diamonds). The right axis represents the relationship between the rates of self-compatibility and inbreeding depression in the same five populations (*R*^2^ = 0.94; squares). Correlation estimates (adjusted *R*^2^) were obtained from linear regression in the statistical software R, version 3.0.1 (R core team. 2013).

Dominance interactions among *S*-alleles that facilitate biparental inbreeding may be a good strategy that allows SI plants to colonize novel habitats without losing SI functionality. A functional SI system is beneficial for progeny fitness in the long term and maintaining this system while overcoming short-term Allee effects could be a strong advantage to invasive plants. We found a large increase in MA relative to MA estimates based on individuals that were fully compatible and therefore not likely to share *S*-alleles. This increase was attributable to dominance interactions among *S*-alleles allowing individuals with one shared allele to successfully mate. The fitness costs associated with inbreeding were highly linear across all life-history stages, with extremely high fitness costs or inbreeding depression incurred from selfing. To assess the trade-off between the increase in MA and inbreeding depression, we assessed the correlation between the proportion of MA due to dominance interactions among *S*-alleles and inbreeding depression estimated separately for each of the five populations (Fig. 5). At the same time, we examined the relationship between inbreeding depression and the rate of self-compatibility in each population (Fig. 5). These comparisons suggest that as inbreeding depression from selfing increases, the contribution to MA from dominance interactions that allow mating among relatives increases. Not surprisingly, as the cost of selfing increased, the rate of self-compatibility decreased.

Wild radish is a highly successful invasive weed that is globally established. Radish experiences severe inbreeding depression for selfed individuals yet mate availability is likely to be extremely limited for colonizing seeds. The seedpod contains several seeds that are often half- or full-siblings (Karron and Marshall 1990; Elam et al. 2007), and the multiple individuals contained in one pod may establish a population if ID from biparental inbreeding is not too severe.

We estimated inbreeding depression for four levels of relatedness within wild radish (*f* = 0, 0.125, 0.250. 0.500) and found a strongly linear and consistent relationship between genetic relatedness and fitness costs (Fig. 3). Although this is to be expected in the absence of epistasis (Crow and Kimura 1970), it is rarely empirically estimated (Stift et al. 2013). Our estimates of cumulative biparental ID are remarkably close to the estimates of biparental inbreeding in the closely related domestic radish, *R. sativus* that is likely derived from *R. raphanistrum*. Nason and Ellstrand (1995) found a cumulative ID of 0.58 for FS and 0.37 for HS which is similar to our respective estimates of 0.61 and 0.36, although the individual life stage estimates of ID in this study vary considerably from theirs. They did not estimate ID for selfed (*f* = 0.5) individuals but predicted ID of 0.9 to 0.99 based on a expected linear relationship between genetic relatedness and ID, which is similar to our estimates. Interestingly, Nason and Ellstrand (1995) found reductions in maternal fecundity drove 94% of the cumulative ID, whereas we found higher rates of ID for seed-set (pregermination) than seedpod production (fecundity), with both life stages contributing significantly to cumulative ID. Notably, for half-siblings, seedpod weight ID was only 0.09 and therefore did not contribute much to cumulative ID.

How does radish successfully invade if mate availability is limited and ID for selfing is so high? Most SI plants have some rate of SC (or PSC) and often the invasion process is a time for evolution from SI to SC (Busch and Schoen 2008). Annual SI plants have been found to exhibit this pattern of higher SI in native ranges and higher SC in invaded ranges (Petanidou et al. 2012). Annual plants do not have multiple years to wait for mates to arrive and populations should suffer from Allee effects or a negative relationship between population density and fitness (Petanidou et al. 2012). However, in wild radish, the cumulative estimates of ID for selfed individuals are likely too severe (ID = 0.95) for purging to occur. In general, ID greater than 50% will prevent the evolution of SC.

Mixed mating (20–80% outcrossing) is common in many taxa and may be an evolutionary stable strategy (Goodwillie et al. 2005; Winn et al. 2011), not just a mid-point on an evolutionary trajectory between SI and SC. The costs of inbreeding depression in mixed mating taxa are similar to that of SI taxa, indicating that purging is not effectively reducing inbreeding depression as the rates of SC increase (Winn et al. 2011). Biparental inbreeding can reduce the cost of selfing by reducing relative inbreeding depression between selfed and outcrossed progeny (Uyenoyama 1986). Biparental inbreeding often occurs in taxa with mixed mating systems (Uyenoyama 1986) and may contribute to the evolution of mixed mating (Griffin and Eckert 2003).

Pollination biology may play a central role in biparental inbreeding (Uyenoyama 1986) as well as in the maintenance of mixed mating systems (Porcher and Lande 2005). Pollen limitation, or the amount of pollen available per ovule (Larson and Barrett 2000), can increase the advantage of selfing as it becomes more efficient to self-pollinate (Porcher and Lande 2005). However, theoretical models predict that the interacting effects of pollen limitation and pollen discounting (the loss of pollen available for outcrossing due to self-pollination) will maintain high rates of selfing while maintaining a selfing rate less than one (Porcher and Lande 2005). Yet, the maintenance of low rates of selfing, as observed in *R. raphanistrum,* cannot be explained by the interactions of pollination biology and inbreeding depression (Porcher and Lande 2005). Therefore, it is likely that the invasion success of *R. raphanistrum* is due to a different strategy.

Elam et al. (2007) investigated Allee effects on maternal fitness for *R. sativus* and found that fitness increased with population size from 2 to 20 individuals, with the smallest populations (n = 2) producing ≈ 25% seed per flower compared with that in the largest populations (n = 20). These results, combined with high cumulative ID of selfed individuals, suggest that *R. raphanistrum* must utilize some mechanism to overcome this Allee effect in establishing populations. Elam et al. (2007) hypothesized that “radishes multiseeded fruits are an apparent adaptation to overcome the challenge of the Allee effect” given each fruit has an average of 3–5 seeds and are often “sired by more than one father”. These authors suggest that seeds from three to four fruits would then be enough to overcome the Allee effect given the effects are strongest with < 10 individuals.

Our results suggest that the dominance system that permits biparental inbreeding is a contributing mechanism that facilitates population establishment in this SSI plant. Individual seedpods contain multiple seeds, but at minimum, the seeds are half-siblings. If seeds from only one or very few seedpods establish a new site, then mating among related individuals is necessary to overcome the Allee effect. We found that mating among related individuals increased mate availability substantially, with the proportion of MA due to dominance interactions ranging from 19 to 46% across five populations (Figs 1, 4). Interestingly, it appears that the contribution of dominance interactions to MA increases as the cost of selfing increases (Fig. 5). We compared the proportion of matings due to dominance interactions in each population with ID (*f* = 0.500; seed-set) and found a strong positive correlation between the contribution of dominance interactions to MA and the cost of selfing (*R*^2^ = 0.62). Additionally, as ID increases, the rate of SC in each population decreases (Fig. 5; *R*^2^ = 0.94), as is expected when populations carry a high genetic load, the cost of being SC is often too high to persist (Husband and Schemske 1996).

Dominance interactions among *S*-alleles in SSI systems have been suggested as a mechanism that may increase mate availability when the number of *S*-alleles are low, such as during the colonization phase of the invasion process (Brennan et al. 2003, 2010; Lafuma and Maurice 2007; Abbott et al. 2008). Brennan et al. (2003) examined the role of dominance interactions at the *S*-locus in *Senecio squalidus*. They speculated that selection pressure for reproductive assurance increases dominance relationships based on the relative proportion of dominance relationships within populations compared with among populations. The relationship between the proportion of MA due to dominance and ID suggests there is selection pressure for increased MA due to mechanisms other than PSC or SC when the cost of SC is high, and therefore, SC rates are low. As a result, dominance interactions become an important mechanism to increase MA.

While GSI is the more common genetic mechanism preventing self-fertilization, SSI is known to occur in at least 10 phylogenetically diverse families of plants, and an additional 47 families have an undetermined genetic mechanism controlling SI, indicating SSI likely occurs in additional plant families (Igic et al. 2008). The widespread nature of SSI suggests *S*-allele dominance interactions could provide reproductive assurance to a range of angiosperms when population size is small and diversity in *S*-alleles is low.

This study empirically documented the increase in MA due to *S*-allele dominance interactions while showing the resulting fitness cost of biparental inbreeding. We found a consistent increase in MA due to dominance interactions as the cost of selfing increased. *R. raphanistrum* is a classic species to document these patterns as it is a highly outcrossing species with an SSI mating system that incurs a very high cost of selfing. Yet, it has been an incredibly successful at invading habitats globally while retaining a functional SI system. The results from this study support the hypothesis that dominance interactions increase MA in SSI plants and demonstrate that biparental inbreeding depression is likely low enough to allow plants to overcome the short-term Allee effects encountered during the colonization process.
